# Reviewed article: Lopes BT, Oliveira CH, Souza PAA, Cordeiro-de-Barros RM, Teixeira TJ, Santos RM, Menezes AS, Abreu FVS. Ocurrence of Nyssomyia intermedia (Diptera: Psychodidae) in intradomiciliary environments: a potential vector of american cutaneous leishmaniasis in Montezuma, 2024. Epidemiol Serv Saude. 2025:34;e20250198

**DOI:** 10.1590/S2237-96222025v34e20250198.a

**Published:** 2025-10-27

**Authors:** Kaio Albarado

**Affiliations:** 1 Universidade Federal do Pará, Belém, PA, Brazil Universidade Federal do Pará Belém PA Brazil

## Peer review A

## Questionnaire

Does the manuscript contain new and significant information to justify publication? Yes

Does the Abstract (Summary) clearly and accurately describe the content of the article? Yes

Is the problem significant and concisely stated? Not applicable

Are the methods described comprehensively? Yes

Are the interpretations and conclusions justified by the results? Yes

Is adequate reference made to other work in the field? Yes

## Manuscript Structure

Length of article is: Adequate

Number of tables is: Adequate

Number of figures is: Adequate

Please state any conflict(s) of interest that you have in relation to the review of this paper. None

Interest: Excellent

Quality: Excellent

Originality: Good

Overall: Excellent

Recommendation: Major Revision

## Comments

Esta pesquisa mostra uma importante relação da atividade humana ao meio ambiente e suas implicações ao contrair uma doença como a Leishmaniose. Porém sugiro pequenas correções nos seguintes itens:

- Métodos: trazer mais detalhes sobre o protocolo descrito por Barreto e Coutinho (linha 11) e sobre a identificação de acordo com Galati (linha 13).

- Discussão: Sugiro retirar o primeiro parágrafo. Parece muito com o que já está nos resultados. Sugiro ainda, expandir a discussão trazendo autores mais atuais, de preferência, dos estudos de 2020 em diante. Esta última sugestão, também se aplica no artigo como um todo. Trazer referências mais atualizadas ao longo do manuscrito.

